# The prevalence of gastric heterotopia of the proximal esophagus is underestimated, but preneoplasia is rare - correlation with Barrett’s esophagus

**DOI:** 10.1186/s12876-017-0644-3

**Published:** 2017-07-12

**Authors:** Ulrich Peitz, Michael Vieth, Matthias Evert, Jovana Arand, Albert Roessner, Peter Malfertheiner

**Affiliations:** 1 Clinic of Gastroenterology, Hepatology, and Infectious Diseases, Otto-von-Guericke University, Leipziger Str. 44, D 30120 Magdeburg, Germany; 2 Clinic of Gastroenterology, Raphaelsklinik, Münster, Germany; 3 Institute of Pathology, Klinikum Bayreuth, Bayreuth, Germany; 4 Institute of Pathology, University Regensburg, Regensburg, Germany; 5 Institute of Pathology, Otto-von-Guericke University, Magdeburg, Germany

**Keywords:** Gastric heterotopia, Inlet patch, Esophagus, Preneoplasia, Intestinal metaplasia, Barrett’s esophagus

## Abstract

**Background:**

The previously reported prevalence of gastric heterotopia in the cervical esophagus, also termed inlet patch (IP), varies substantially, ranging from 0.18 to 14%. Regarding cases with adenocarcinoma within IP, some experts recommend to routinely obtain biopsies from IP for histopathology. Another concern is the reported relation to Barrett’s esophagus. The objectives of the study were to prospectively determine the prevalence of IP and of preneoplasia within IP, and to investigate the association between IP and Barrett’s esophagus.

**Methods:**

372 consecutive patients undergoing esophagogastroduodenoscopy were carefully searched for the presence of IP. Biopsies for histopathology were targeted to the IP, columnar metaplasia of the lower esophagus, gastric corpus and antrum. Different definitions of Barrett’s esophagus were tested for an association with IP.

**Results:**

At least one IP was endoscopically identified in 53 patients (14.5%). Histopathology, performed in 46 patients, confirmed columnar epithelium in 87% of cases, which essentially presented corpus and/or cardia-type mucosa. Intestinal metaplasia was detected in two cases, but no neoplasia. A previously reported association of IP with Barrett’s esophagus was weak, statistically significant only when short segments of cardia-type mucosa of the lower esophagus were included in the definition of Barrett’s esophagus.

**Conclusions:**

The prevalence of IP seems to be underestimated, but preneoplasia within IP is rare, which does not support the recommendation to regularly obtain biopsies for histopathology. Biopsies should be targeted to any irregularities within the heterotopic mucosa. The correlation of IP with Barrett’s esophagus hints to a partly common pathogenesis.

**Electronic supplementary material:**

The online version of this article (doi:10.1186/s12876-017-0644-3) contains supplementary material, which is available to authorized users.

## Background

Islands of gastric mucosa in the proximal esophagus are commonly designated as inlet patches (IP). They are considered to be heterotopic in nature in that they represent remnants of the columnar lining of the fetal esophagus. Discussed sequelae of clinical significance are laryngitis, esophagitis, esophageal web, stricture, ulcer, perforation, fistula or adenocarcinoma [1]. Severe sequelae are rare, reported only in individual case reports. More than fifty cases of adenocarcinoma arising from an IP have been reported between 1950 and 2016 (literature reviews [2, 3], recent case reports [2, 4–12]). Some experts recommend to take biopsies from IP in order to detect neoplastic or preneoplastic alterations [13–15], or advise follow-up examinations [15, 16]. Before such recommendations can be generalized, more data on the prevalence of preneoplastic alterations in IP are needed.

Data on the very prevalence of IP diverge a lot. Prospective studies have yielded higher prevalences, ranging from 1 to 14% [15, 17–33], than studies with retrospective design, 0.18 to 1.6% [13, 17, 34–41].

Preneoplastic conditions or lesions of IP are not yet defined. IP may contain any type of mucosa of the normal stomach, i.e. antrum, corpus or cardia mucosa, but also intestinal metaplasia [15, 19, 21, 26, 27, 31, 35, 38, 39, 41, 42]. In the stomach, *Helicobacter pylori* infection, mucosal atrophy and intestinal metaplasia (Correa cascade) increase the risk of gastric adenocarcinoma [43, 44]. In the lower esophagus, Barrett’s esophagus is an established preneoplasia. But there are differing definitions of Barrett’s esophagus regarding the type of columnar metaplasia. The risk for esophageal adenocarcinoma is lower with pure gastric metaplasia than with intestinal metaplasia. Among others, American and German guidelines require the presence of intestinal metaplasia to define Barrett’s esophagus [45–47]. Retrospective publications have indicated that there is an association between the presence of IP and that of Barrett’s esophagus [25, 29, 35, 36, 38, 48] or even adenocarcinoma of the lower esophagus [38, 48].

The aims of the study were to determine the prevalence of IP in a prospective endoscopic study, to characterize the type of columnar epithelium within these IP, in particular with respect to preneoplastic conditions, and to investigate the association between IP and Barrett’s esophagus.

## Methods

### Patients

The study was based on patients referred for esophagogastroduodenoscopy (EGD) to the endoscopy unit of the University Magdeburg. Prior to starting the prospective study, the prevalence of IP was determined retrospectively. By searching the electronic files from January 1996 through January 2002, fifty patients with endoscopic description of IP were retrieved out of 9928 EGD, corresponding to a frequency of 0.5%.

The prospective study lasted from February to June 2002. It was approved by the Ethics Committee of our university and conformed to the provisions of the Declaration of Helsinki. Patients gave written informed consent. During the five months period, the prevalence of IP was determined in consecutive patients endoscopically examined by one investigator (UP). Of the 444 EGDs he performed the following were excluded: emergency cases (*n* = 31), percutaneous endoscopic gastrostomy (*n* = 5), patients after esophagus resection (*n* = 4), malignant tumor (*n* = 2) or severe esophagitis (*n* = 4) in the proximal part of the esophagus, repetitive endoscopies during the study period (*n* = 26). All other patients with consent (*n* = 372) were included, irrespective of the indication. For statistical analysis, indications were dichotomized into dominant reflux symptoms (*n* = 93) versus the remainder (*n* = 279). Outpatients were 175, inpatients 197.

### Endoscopy

EGD was performed using routine video-endoscopes (GIF Q 145, Olympus Optical, Hamburg, Germany). An endoscopic diagnosis of IP was made when an island of salmon red velvety mucosa was identified in the proximal esophagus. The number of IP and the maximum diameter of the largest IP were documented. An attempt was made to take biopsies from IP for histological evaluation in any patient concerned, but contraindications against biopsies or technical difficulties in taking biopsies were respected and documented. Biopsies from gastric antrum and corpus were obtained according to the updated Sydney protocol [49].

Any pathology revealed by EGD was documented, as was conscious sedation, mainly midazolam iv, partly in combination with pethidin iv. The quality of visualization of the esophageal mucosa was graded into a 3-point scale.

Any columnar epithelium extending more than 0.5 cm proximal to the esophago-gastric junction, be it in form of tongues, islands or circumferential areas, were documented as “columnar epithelium lined lower esophagus” (CLE) and, if not contraindicated, biopsied for histopathology according to guidelines on Barrett’s esophagus [50, 51]. The length of CLE and the maximum diameter of IP were estimated using an open biopsy forceps, or, in cases with long segments, comparing the distance from the incisors.

### Histopathology

Histological slides were stained with hematoxilin-eosin and a modified Giemsa stain (2%) to detect *Helicobacter pylori* bacteria. In cases with doubtful *Helicobacter pylori* status, Warthin-Starry stain was used in addition. Detection of goblet cells led to diagnosis of intestinal metaplasia.

### Categories of Barrett’s esophagus

Based on the endoscopically determined length of CLE and the histopathological detection of columnar epithelium with or without intestinal metaplasia, four categories with different definitions of Barrett’s esophagus were tested as independent variables: (1) CLE of at least 0.5 cm length, any columnar epithelium; (2) CLE of at least 3 cm length, any columnar epithelium; (3) CLE of at least 0.5 cm length, columnar epithelium with intestinal metaplasia, (4) CLE of at least 3 cm length, columnar epithelium with intestinal metaplasia.

### Statistics

The expected prevalence of IP was estimated at 6%, based on the average of previous data of prospective studies in the literature. To achieve a width of 5% for the 95%-confidence interval of the proportion of patients with IP (prevalence), a sample size of *n* = 350 was calculated. The differences between the proportions of cases with and without IP in relation to different independent variables were statistically analyzed using non-parametric tests. Two-sided *P* values of less than 0.05 were considered to be statistically significant. The statistical software used was IBM SPSS Statistics 24™.

## Results

**Fig. 1:**
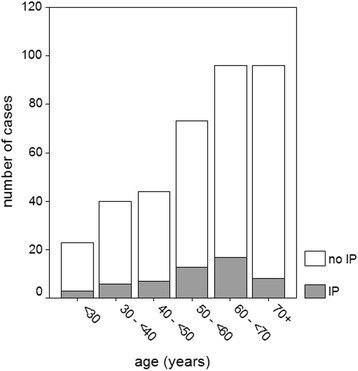
Number of cases with and without inlet patch (IP) in relation to age categories

Out of the 372 patients included, at least one IP was identified endoscopically in 54 cases (14.5%, 95% confidence interval 10.9%–18.1%). Demographic data are shown in Table 1. More males than females had IPs detected (18 vs 11%), but the difference was not statistically significant. There was an insignificant trend for a higher prevalence of IP between 50 and 70 years of age compared to the prevalence in younger or older subjects (Fig. 1).

**Table 1:** Demographic data of patients with and without IP

	Patients with IP	Patients without IP	Level of statistical significance
Number	*n* = 54	*n* = 318	
Gender, number (percentage of columns)			Chi square test *p* = 0.09
Female	*n* = 21(39%)	*n* = 163 (49%)	
Male	*n* = 33 (61%)	*n* = 155 (51%)	
Age (years)			Mann-Whitney test *p* = 0.44
Minimum	19	18	
Median	57	60	
Maximum	89	93

All IPs were located in the cervical part of the esophagus, mostly within 1 to 5 cm distal to the upper esophageal sphincter, but partly also at the level of the sphincter. Although the detection rate of IP increased with the grade of visualization, this correlation was not significant. There was no correlation with the use of conscious sedation.

**Fig. 2:**
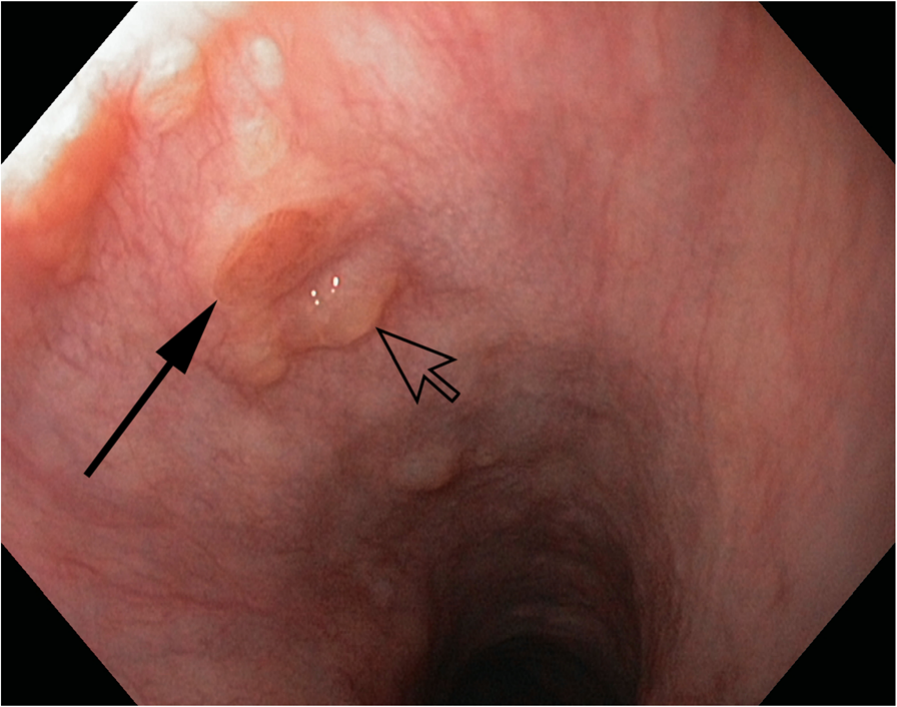
Endoscopic view of a small inlet patch (*solid arrow*), estimated 0.3 cm in diameter, surrounded by subsquamous glands (*yellow spots*), including a cyst (*open arrow*)

**Fig. 3:**
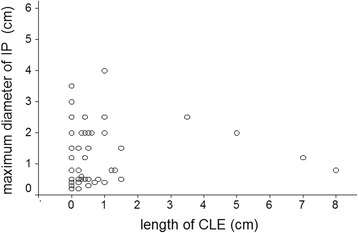
Scatter diagram of length of inlet patch (IP) in relation to length of columnar epithelium lined lower esophagus (CLE)

A single IP was observed in 37 cases. In 17 patients there were multiple IPs; 2 of them in 9; 3 IPs in 5; and 5 to 7 IPs in 3 cases. The maximum diameter of the largest IP ranged from 0.2 to 4 cm (Fig. 2). A scatter diagram of the maximum diameter is shown in Fig. 3.

**Fig. 4:**
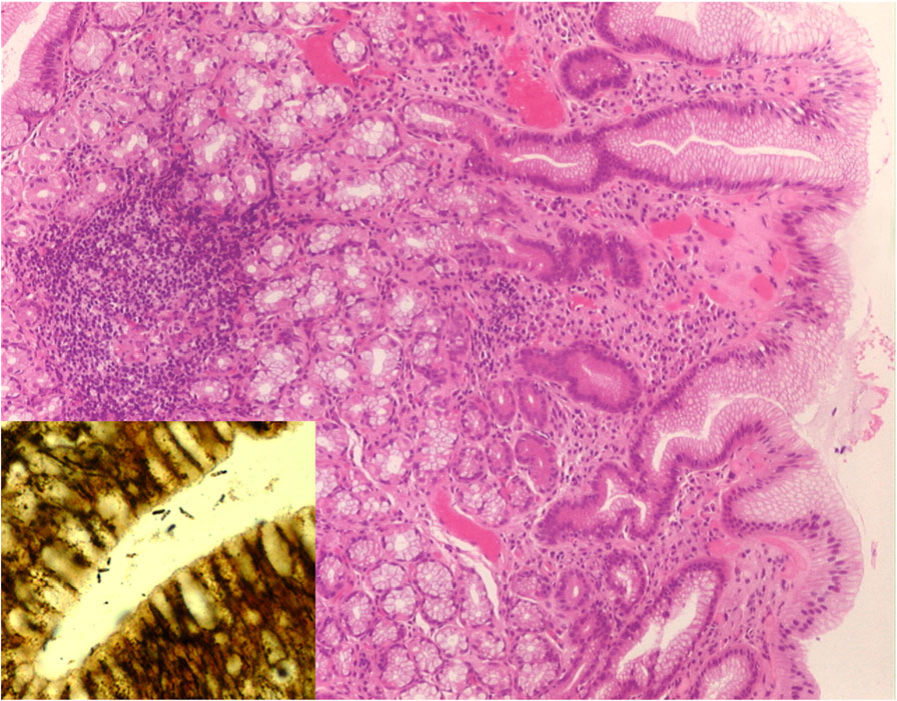
Histological view of a biopsy from a 0.5 cm inlet patch with predominating cardia-type glands. Moderate chronic and mild active inflammatory infiltrate associated with *Helicobacter pylori* infection (*inset*). Hematoxylin & eosin, original magnification ×100; Inset: Whartin Starry stain, original magnification ×1000

**Fig. 5:**
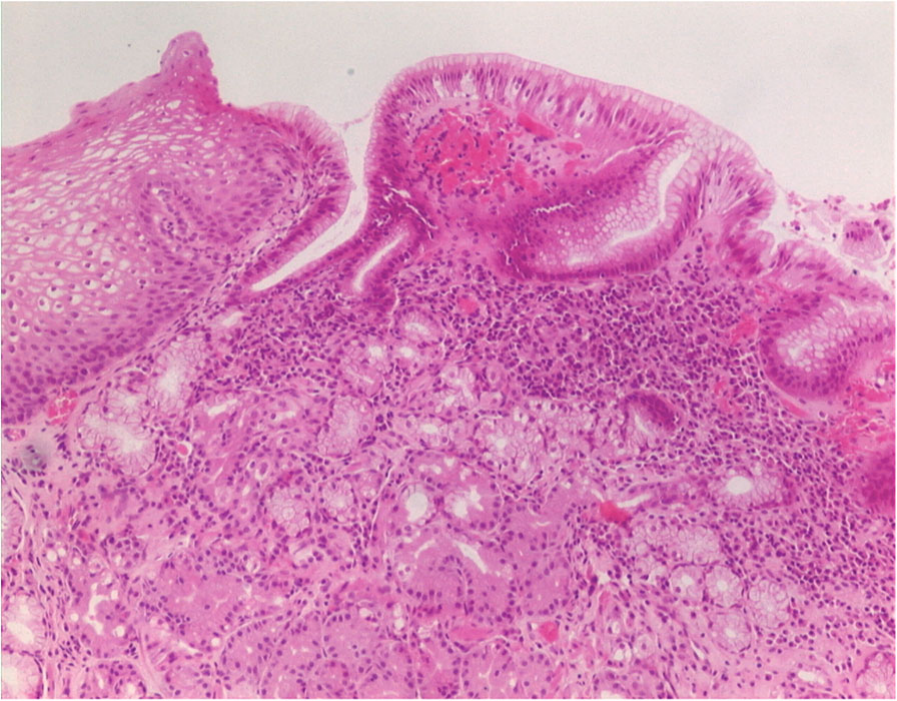
Histological view of a biopsy from a 12 mm inlet patch with transition of normal esophageal squamous epithelium (*left*) into gastric foveolar epithelium with cardia-type glands and deeper located corpus glands. Moderate chronic inflammation without *Helicobacter pylori* infection. Hematoxylin & eosin, original magnification ×100

In 46 patients with visible IP, at least one biopsy could be targeted to the IP (1 biopsy in *n* = 7; 2 biopsies in *n* = 24; 3 in *n* = 14; and 5 in *n* = 1 patients). Reasons for not obtaining biopsies were uneasiness or retching in 6, and coagulation disorders in 2 patients. Columnar epithelium could be confirmed in *n* = 40 (87%) of these patients. Cardia- and corpus-type mucosa were found at an almost equal frequency (Table 2). Biopsies from small IPs tended to contain more often cardia-type mucosa (Fig. 4), while those from larger IPs were more frequently composed of corpus mucosa. At the border between columnar and squamous cell epithelium, cardia-type mucosa was the predominant type of columnar mucosa (Fig. 5).

**Table 2:** Histology of IP in all patients with biopsy targeted to IP, and separately in two subgroups stratified according to maximum diameter of IP (percentages of columns)

	All patients with biopsy, *n* = 46	Maximum diameter of IP	
		<1 cm, *n* = 24	≥1 cm, *n* = 22
	Number (%)	Number (%)	Number (%)
Cardia mucosa	16 (35%)	10 (42%)	6 (27%)
Cardia plus corpus mucosa	12 (26%)	7 (29%)	5 (23%)
Corpus mucosa	12 (26%)	4 (17%)	8 (36%)
Only squamous epithelium	6 (13%)	3 (12%)	3 (14%)

**Fig. 6:**
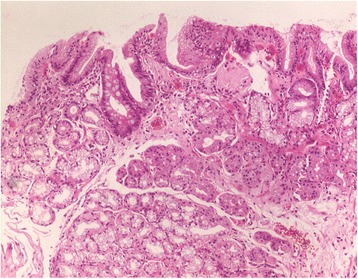
Histological view of a biopsy from a 2.5 cm inlet patch with a combination of mucoid cardia-type and corpus-type glands and superficial focal intestinal metaplasia. Few subepithelial lymphocytes and plasma cells indicate a very mild chronic inflammatory reaction. Hematoxylin & eosin, original magnification ×100

There were 277 patients with a complete set of biopsies from the gastric antrum and corpus, and, if endoscopically detected, from IP. The prevalence of IP in this subgroup was *n* = 43 (15.5%) by endoscopy, and *n* = 36 (12.9%) confirmed by histopathology. In gastric biopsies, *Helicobacter pylori* bacteria were detected in *n* = 45 (16%). Five of these were found with an IP, but only one had *Helicobacter pylori* detected also in his IP (Fig. 4). Overall, mild chronic inflammation of IP was present in 37, moderate in 4 cases (Figs. 4, 5 and 6). Active inflammatory infiltration of IP was always mild and occurred in 8 patients, including the one with *Helicobacter pylori*. The presence of chronic or active inflammation of IP was correlated neither with the presence of *Helicobacter pylori* infection of the stomach nor with any of the gastroesophageal reflux parameters mentioned below.

Within IP, we observed intestinal metaplasia in two cases (Fig. 6). One 59-year-old patient had a single IP of 0.5 cm diameter, the other, aged 65 years, a single IP of 2.5 cm. Both were males with reflux symptoms, but without CLE. In both cases intestinal metaplasia was focal. Only the latter patient exhibited focal intestinal metaplasia also in the corpus.

The relationship between IP and parameters of gastroesophageal reflux are shown in Table 3. Although the prevalence of IP was higher in patients with dominant reflux symptoms, hiatal hernia, reflux esophagitis, or Barrett’s esophagus than in those without these respective conditions, these relations were not statistically significant. Only the higher prevalence of IP in patients with a CLE of at least 0.5 cm length and any columnar epithelium on histology was significant (*p* = 0.02, odds ratio 2.1). There was no significant correlation between the grade of reflux esophagitis and the presence of IP, nor between the length of CLE and the presence of IP, and nor between the length of CLE and the maximum diameter of IP (Fig. 3).

**Table 3:** Relation of the prevalence of IP with gastroesophageal reflux parameters and with different categories of Barrett’s esophagus

	Prevalence of IP in the condition	Prevalence of the condition in patients with IP	Prevalence of the condition in patients without IP	Level of statistical significance (Fisher’s exact test, two-sided)
	Number (%)	Number (%)	Number (%)	
Dominant reflux symptoms	15/93 (16%)	15 (27%)	78 (24%)	*p* = 0.61
Hiatal hernia	13/83 (16%)	13 (24%)	70 (22%)	*p* = 0.74
Reflux esophagitis	14/75 (19)%	14 (26%)	61 (19%)	*p* = 0.25
CLE ≥ 0.5 cm	21/95 (22%)	21 (39%)	74 (23%)	*p* = 0.02
CLE ≥ 3 cm	4/18 (22)%	4 (7%)	14 (4%)	*p* = 0.31
CLE ≥ 0.5 cm with IM	7/30 (23%)	7 (13%)	23 (7%)	*p* = 0.15
CLE ≥ 3 cm with IM	3/14 (21%)	3 (6)%	11 (4%)	*p* = 0.44

*CLE* columnar epithelium lined lower esophagus (endoscopic diagnosis)

*IM* intestinal metaplasia (histopathologic diagnosis)

In the total study sample, the following pathological findings were documented in addition to those of Table 3: esophageal thrush 3%, esophageal tumor 1%, esophageal varices 7%, esophageal peptic stenosis 1%, gastric ulcer 7%, gastric erosions 16%, gastric tumor 2%, previous distal gastric resection 2%, duodenal ulcer 2%, duodenal erosions 2%, duodenal stenosis 1%. None of these were significantly related to IP. A complete normal finding in the upper gastrointestinal tract was observed in 201 (54%) cases.

## Discussion

The 14.5% prevalence of IPs revealed in this study is the highest ever reported as an English full text of a clinical study, to the best of our knowledge. But some studies report prevalences close to this, 10% by Borhan-Manesh et al. [18], 11% by Weickert et al. [27], 12% by Chung et al. [30] using narrow band imaging, 13% by Vesper et al. [33] and 14% by Kumagai et al. [23]. In an abstract, Ohara et al. [52] report even 21%, also using narrow band imaging. The same prevalence of 21% was yielded by an autopsy series of infants and children [53]. In contrast, in the retrospective part of our study, the prevalence was low (0.5%), within the range of previously reported retrospective studies (0.18 to 1.6%) [13, 17, 34–41]. The discrepancy between retrospective and prospective studies is a clear indication that retrospective data comprise endoscopies in which IPs were often overlooked or neglected.

Nevertheless, IP should be looked for, and, if present, mentioned in examination reports. IP may give rise to benign or malignant sequelae, even though very rarely. Recent case reports on adenocarcinoma in IP include cases with small and flat lesions, fairly discernible from benign IP [6, 11]. Furthermore, IP should be distinguished from early esophageal squamous neoplasia, which also exhibits a flat red discoloration.

Like in the stomach and in Barrett’s esophagus, intestinal metaplasia of IP may represent a preneoplastic condition. It has been described to occur in conjunction with an adenocarcinoma of IP [10, 54–56]. However, there are no long-term data on the risk of neoplasia emerging from intestinal metaplasia of IP. In those 40 cases with histologically confirmed IP of our study, the prevalence of intestinal metaplasia was *n* = 2 (5%), admittedly a number too small to representatively estimate the proportion. This proportion is of similar magnitude as in other studies reporting intestinal metaplasia in IP, ranging from 0 to 12% [15, 19, 21, 26, 27, 31, 35, 38, 39, 41, 42]. The two largest studies provided proportions of 1% [38] respectively 3% [39].

The high prevalence of IP in relation to the limited number of published cases with adenocarcinoma originating from IPs [2–12] challenges the recommendation given by some experts to obtain biopsies for histopathology from any IP [13–15]. Furthermore, taking biopsies in the proximal esophagus often provokes retching or coughing which makes it an uncomfortable or even risky approach. In our series, the low proportion of cases with intestinal metaplasia and the lack of any neoplasia within IP do not support such a recommendation. However, any irregularity of the mucosal surface of an IP identified on endoscopic examination should prompt taking targeted biopsies.

An high association of IP with Barrett’s esophagus was reported in previous publications [25, 29, 35, 36, 38, 48], but not confirmed by other studies [13, 18, 57]. We tested four different definitions of Barrett’s esophagus for an association with IP. The reason is that in different guidelines there are conflicting definitions with respect to the histopathological verification of Barrett’s esophagus. Some guidelines consider the presence of intestinal metaplasia as mandatory [45–47], whereas others require only columnar epithelium [50, 58]. Cardia-type mucosa in the lower esophagus has consistently been shown to be an acquired type of mucosa [59–61], and is likely to be a precursor of intestinal metaplasia [62] and of adenocarcinoma as well [63]. Therefore, we took into account also cases with endoscopically detected columnar lining in the lower esophagus (“CLE”) that exhibited only cardia-type mucosa on histological evaluation, but no intestinal metaplasia. Only for this category of CLE, there was a significant association with IP.

One limitation of the study is that patients from a tertiary referral center were examined rather than a sample from the general population. Certainly, such patients are not representative of the general population, but currently there are no data to give consistent evidence that IPs might be significantly correlated with any other pathology, except for the relation with Barrett’s esophagus.

Another limitation is the delay between the study and its publication. Advanced endoscopic modalities like high density resolution, near focus and virtual chromoendoscopy were not yet applicable, but might ameliorate the detection rate of IP. However, Vesper et al. [33] found a high prevalence of IP (13.3%), which was comparable and not significantly different among standard definition videoendoscopy (12.7%), high definition endoscopy (14.4%), and narrow-band imaging (14.2%).

Endoscopic diagnosis of IP was confirmed by histopathology in 87%, which reduces the prevalence of IP to 12.6% as calculated for the whole study sample, or to 12.9% as counted in the subgroup of cases with a complete set of biopsies from stomach and esophagus. The most probable explanation for cases with endoscopic diagnosis of IP, but without histological confirmation, is unsuccessful targeting of the biopsy to a very small IP.

Half of our patients with IP had a maximum diameter of the largest IP of less than 1 cm (Table 2). The small IPs were mostly composed of cardia-type mucosa, whereas the larger ones were more likely to contain corpus mucosa centrally. In the vicinity of IPs within squamous epithelium, one frequently observes yellow spots (Fig. 2). These were not taken into account as IP in our study. They contain foci of subsquamous columnar epithelium, addressed by some pathologists as esophageal glands proper. Noteworthy, these yellow spots resemble those in squamous epithelium close to the squamocolumnar junction of the esophagogastric junction [64]. Esophageal submucosal glands are known to be clustered at either end of the esophagus [65]. A convincing though unproven concept is, that such foci represent a precursor of columnar metaplasia of the esophagus [66]. According to this concept, intraepithelial cysts erupt to the surface to build the columnar metaplasia. Our observation of very small IPs on the top of such yellow spots support the existence of such a dynamic process (Fig. 2).

## Conclusions

The prevalence of IPs is often underestimated because IP may be overlooked or neglected. Regular biopsies for histopathology from any IP cannot be recommended because preneoplasia within IP is rare. Careful endoscopic inspection of IP, however, seems to be worthwhile in order to detect early malignancy and to differentiate IP from squamous cell neoplasia. The relation of IP with Barrett’s esophagus, though clinically of minor relevance, may stimulate research on the common pathogenesis of IP and Barrett’s esophagus.

## Additional file

Additional file 1: Gastric Heterotopia datasets. Contains demographic, endoscopic and histological data, as mentioned at the head of columns. (XLSX 53 kb)

## Abbreviations

CLE: columnar epithelium lined lower esophagus
IM: intestinal metaplasia
IP: Inlet patch

## References

[1] von Rahden BH, Stein HJ, Becker K, Liebermann-Meffert D, Siewert JR (2004). Heterotopic gastric mucosa of the esophagus: literature-review and proposal of a clinicopathologic classification. Am J Gastroenterol.

[2] Kitajima T, Kaida S, Lee S, Haruta S, Shinohara H, Ueno M, Suyama K, Oota Y, Fujii T, Udagawa H (2013). Mixed adeno(neuro)endocrine carcinoma arising from the ectopic gastric mucosa of the upper thoracic esophagus. World J Surg Oncol.

[3] Komori S, Osada S, Tanaka Y, Takahashi T, Nagao N, Yamaguchi K, Asano N, Yoshida K (2010). A case of esophageal adenocarcinoma arising from the ectopic gastric mucosa in the thoracic esophagus. Rare Tumors.

[4] Kadota T, Fujii S, Oono Y, Imajoh M, Yano T, Kaneko K (2016). Adenocarcinoma arising from heterotopic gastric mucosa in the cervical esophagus and upper thoracic esophagus: two case reports and literature review. Expert Rev Gastroenterol Hepatol.

[5] Hudspeth VR, Smith DS, Pacicco T, Lewis JJ (2016). Successful endoscopic resection of adenocarcinoma arising in an esophageal inlet patch. Dis Esophagus.

[6] Probst A, Schaller T, Messmann H: Adenocarcinoma arising from ectopic gastric mucosa in an esophageal inlet patch: treatment by endoscopic submucosal dissection. Endoscopy 2015, 47 Suppl 1 UCTN:E337–338. 10.1055/s-0034-1392423 26134434

[7] Nomura K, Iizuka T, Inoshita N, Kuribayashi Y, Toba T, Yamada A, Yamashita S, Furuhata T, Kikuchi D, Matsui A (2015). Adenocarcinoma of the cervical esophagus arising from ectopic gastric mucosa: report of two cases and review of the literature. Clin J Gastroenterol.

[8] Ajmal S, Young JS, Ng T (2015). Adenocarcinoma arising from cervical esophageal gastric inlet patch. J Thorac Cardiovasc Surg.

[9] Yasar B, Tarcin O, Benek D, Goksel S (2014). Intramucosal adenocarcinoma arising from ectopic gastric mucosa in the upper esophagus treated successfully with endoscopic mucosal resection. J Gastrointest Cancer.

[10] Tanaka M, Ushiku T, Ikemura M, Shibahara J, Seto Y, Fukayama M (2014). Esophageal adenocarcinoma arising in cervical inlet patch with synchronous Barrett's esophagus-related dysplasia. Pathol Int.

[11] Möschler O, Vieth M, Müller MK: Endoscopic resection of an adenocarcinoma occurring in ectopic gastric mucosa within the proximal esophagus. Endoscopy 2014, 46 Suppl 1 UCTN:E24–25. 10.1055/s-0033-1358807 24523165

[12] Verma YP, Chauhan AK, Sen R (2013). Primary adenocarcinoma of the upper oesophagus. Ecancermedicalscience.

[13] Akbayir N, Alkim C, Erdem L, Sokmen HM, Sungun A, Basak T, Turgut S, Mungan Z (2004). Heterotopic gastric mucosa in the cervical esophagus (inlet patch): endoscopic prevalence, histological and clinical characteristics. J Gastroenterol Hepatol.

[14] Klaase JM, Lemaire LC, Rauws EA, Offerhaus GJ, van Lanschot JJ (2001). Heterotopic gastric mucosa of the cervical esophagus: a case of high-grade dysplasia treated with argon plasma coagulation and a case of adenocarcinoma. Gastrointest Endosc.

[15] Poyrazoglu OK, Bahcecioglu IH, Dagli AF, Ataseven H, Celebi S, Yalniz M (2009). Heterotopic gastric mucosa (inlet patch): endoscopic prevalence, histopathological, demographical and clinical characteristics. Int J Clin Pract.

[16] Abe T, Hosokawa M, Kusumi T, Kusano M, Hokari K, Kagaya H, Watanabe A, Fujita M, Sasaki S (2004). Adenocarcinoma arising from ectopic gastric mucosa in the cervical esophagus. Am J Clin Oncol.

[17] Azar C, Jamali F, Tamim H, Abdul-Baki H, Soweid A (2007). Prevalence of endoscopically identified heterotopic gastric mucosa in the proximal esophagus: endoscopist dependent?. J Clin Gastroenterol.

[18] Borhan-Manesh F, Farnum JB (1991). Incidence of heterotopic gastric mucosa in the upper oesophagus. Gut.

[19] Gutierrez O, Akamatsu T, Cardona H, Graham DY, El Zimaity HM (2003). Helicobacter pylori and hetertopic gastric mucosa in the upper esophagus (the inlet patch). Am J Gastroenterol.

[20] Jabbari M, Goresky CA, Lough J, Yaffe C, Daly D, Cote C (1985). The inlet patch: heterotopic gastric mucosa in the upper esophagus. Gastroenterology.

[21] Jacobs E, Dehou MF (1997). Heterotopic gastric mucosa in the upper esophagus: a prospective study of 33 cases and review of literature. Endoscopy.

[22] Maconi G, Pace F, Vago L, Carsana L, Bargiggia S, Porro GB (2000). Prevalence and clinical features of heterotopic gastric mucosa in the upper oesophagus (inlet patch). Eur J Gastroenterol Hepatol.

[23] Kumagai Y (2005). Incidence of heterotopic gastric mucosa in the upper esophagus (inlet patch). Progr Dig Endosc.

[24] Korkut E, Bektas M, Savas B, Memmedzade F, Oztas E, Ustun Y, Idilman R, Ozdena A (2009). Awareness of the endoscopist affects detection rate of heterotopic gastric mucosa in esophagus. Indian J Gastroenterol.

[25] Yuksel I, Uskudar O, Koklu S, Basar O, Gultuna S, Unverdi S, Ozturk ZA, Sengul D, Arikok AT, Yuksel O (2008). Inlet patch: associations with endoscopic findings in the upper gastrointestinal system. Scand J Gastroenterol.

[26] Alagozlu H, Simsek Z, Unal S, Cindoruk M, Dumlu S, Dursun A (2010). Is there an association between helicobacter pylori in the inlet patch and globus sensation?. World J Gastroenterol.

[27] Weickert U, Wolf A, Schroder C, Autschbach F, Vollmer H (2011). Frequency, histopathological findings, and clinical significance of cervical heterotopic gastric mucosa (gastric inlet patch): a prospective study in 300 patients. Dis Esophagus.

[28] Cheng CL, Lin CH, Liu NJ, Tang JH, Kuo YL, Tsui YN (2014). Endoscopic diagnosis of cervical esophageal heterotopic gastric mucosa with conventional and narrow-band images. World J Gastroenterol.

[29] Al-Mammari S, Selvarajah U, East JE, Bailey AA, Braden B (2014). Narrow band imaging facilitates detection of inlet patches in the cervical oesophagus. Dig Liver Dis.

[30] Chung CS, Lin CK, Liang CC, Hsu WF, Lee TH (2015). Intentional examination of esophagus by narrow-band imaging endoscopy increases detection rate of cervical inlet patch. Dis Esophagus.

[31] Sahin G, Adas G, Koc B, Akcakaya A, Dogan Y, Goksel S, Yalcin O (2014). Is cervical inlet patch important clinical problem?. Int J Biomed Sci.

[32] Govani SM, Metko V, Rubenstein JH (2015). Prevalence and risk factors for heterotopic gastric mucosa of the upper esophagus among men undergoing routine screening colonoscopy. Dis Esophagus.

[33] Vesper I, Schmiegel W, Brechmann T (2015). Equal detection rate of cervical heterotopic gastric mucosa in standard white light, high definition and narrow band imaging endoscopy. Z Gastroenterol.

[34] Feurle GE, Helmstaedter V, Buehring A, Bettendorf U, Eckardt VF (1990). Distinct immunohistochemical findings in columnar epithelium of esophageal inlet patch and of Barrett's esophagus. Dig Dis Sci.

[35] Avidan B, Sonnenberg A, Chejfec G, Schnell TG, Sontag SJ (2001). Is there a link between cervical inlet patch and Barrett's esophagus?. Gastrointest Endosc.

[36] Tang P, McKinley MJ, Sporrer M, Kahn E (2004). Inlet patch: prevalence, histologic type, and association with esophagitis, Barrett esophagus, and antritis. Arch Pathol Lab Med.

[37] Chen YR, Wu MM, Nan Q, Duan LP, Miao YL, Li XY (2012). Heterotopic gastric mucosa in the upper and middle esophagus: 126 cases of gastroscope and clinical characteristics. Hepato-Gastroenterology.

[38] Neumann WL, Lujan GM, Genta RM (2012). Gastric heterotopia in the proximal oesophagus ("inlet patch"): association with adenocarcinomas arising in Barrett mucosa. Dig Liver Dis.

[39] Fang Y, Chen L, Chen DF, Ren WY, Shen CF, Xu Y, Xia YJ, Li JW, Wang P, Zhang AR (2014). Prevalence, histologic and clinical characteristics of heterotopic gastric mucosa in Chinese patients. World J Gastroenterol.

[40] Rodriguez-Martinez A, Salazar-Quero JC, Tutau-Gomez C, Espin-Jaime B, Rubio-Murillo M, Pizarro-Martin A (2014). Heterotopic gastric mucosa of the proximal oesophagus (inlet patch): endoscopic prevalence, histological and clinical characteristics in paediatric patients. Eur J Gastroenterol Hepatol.

[41] Yu L, Yang Y, Cui L, Peng L, Sun G (2014). Heterotopic gastric mucosa of the gastrointestinal tract: prevalence, histological features, and clinical characteristics. Scand J Gastroenterol.

[42] Bogomoletz WV, Geboes K, Feydy P, Nasca S, Ectors N, Rigaud C (1988). Mucin histochemistry of heterotopic gastric mucosa of the upper esophagus in adults: possible pathogenic implications. Hum Pathol.

[43] Meining A, Bayerdorffer E, Muller P, Miehlke S, Lehn N, Holzel D, Hatz R, Stolte M (1998). Gastric carcinoma risk index in patients infected with helicobacter pylori. Virchows Arch.

[44] Uemura N, Okamoto S, Yamamoto S, Matsumura N, Yamaguchi S, Yamakido M, Taniyama K, Sasaki N, Schlemper RJ (2001). Helicobacter pylori infection and the development of gastric cancer. N Engl J Med.

[45] Spechler SJ, Sharma P, Souza RF, Inadomi JM, Shaheen NJ (2011). American Gastroenterological Association medical position statement on the management of Barrett's esophagus. Gastroenterology.

[46] Koop H, Fuchs KH, Labenz J, Lynen Jansen P, Messmann H, Miehlke S, Schepp W, Wenzl TG (2014). S2k guideline: gastroesophageal reflux disease guided by the German Society of Gastroenterology: AWMF register no. 021–013. Z Gastroenterol.

[47] Shaheen NJ, Falk GW, Iyer PG, Gerson LB: ACG clinical guideline: diagnosis and Management of Barrett's esophagus. Am J Gastroenterol 2016, 111(1):30–50; quiz 51. 10.1038/ajg.2015.322 PMC10245082 26526079

[48] Malhi-Chowla N, Ringley RK, Wolfsen HC (2000). Gastric metaplasia of the proximal esophagus associated with esophageal adenocarcinoma and Barrett's esophagus: what is the connection? Inlet patch revisited. Dig Dis.

[49] Dixon MF, Genta RM, Yardley JH, Correa P (1996). Classification and grading of gastritis. The updated Sydney system. International workshop on the histopathology of gastritis, Houston 1994. Am J Surg Pathol.

[50] Boyer J, Robaszkiewicz M (2000). Guidelines of the French Society of Digestive Endoscopy: monitoring of Barrett's esophagus. The Council of the French Society of digestive endoscopy. Endoscopy.

[51] Sampliner RE (2002). Updated guidelines for the diagnosis, surveillance, and therapy of Barrett's esophagus. Am J Gastroenterol.

[52] Ohara M (2010). Incidence of heterotopic gastric mucosa in the upper esophagus in first time narrow banding image endoscopy of consecutive 900 patients. Gastrointest Endosc.

[53] Variend S, Howat AJ (1988). Upper oesophageal gastric heterotopia: a prospective necropsy study in children. J Clin Pathol.

[54] Christensen WN, Sternberg SS (1987). Adenocarcinoma of the upper esophagus arising in ectopic gastric mucosa. Two case reports and review of the literature. Am J Surg Pathol.

[55] Lauwers GY, Scott GV, Vauthey JN (1998). Adenocarcinoma of the upper esophagus arising in cervical ectopic gastric mucosa: rare evidence of malignant potential of so-called "inlet patch". Dig Dis Sci.

[56] Chatelain D, Lajarte-Thirouard AS, Tiret E, Flejou JF (2002). Adenocarcinoma of the upper esophagus arising in heterotopic gastric mucosa: common pathogenesis with Barrett's adenocarcinoma?. Virchows Arch.

[57] Van Asche C, Rahm AE, Goldner F, Crumbaker D (1988). Columnar mucosa in the proximal esophagus. Gastrointest Endosc.

[58] Fitzgerald RC, di Pietro M, Ragunath K, Ang Y, Kang JY, Watson P, Trudgill N, Patel P, Kaye PV, Sanders S (2014). British Society of Gastroenterology guidelines on the diagnosis and management of Barrett's oesophagus. Gut.

[59] Chandrasoma PT, Der R, Dalton P, Kobayashi G, Ma Y, Peters J, Demeester T (2001). Distribution and significance of epithelial types in columnar-lined esophagus. Am J Surg Pathol.

[60] Chandrasoma PT, Der R, Ma Y, Peters J, Demeester T (2003). Histologic classification of patients based on mapping biopsies of the gastroesophageal junction. Am J Surg Pathol.

[61] Peitz U, Vieth M, Pross M, Leodolter A, Malfertheiner P (2004). Cardia-type metaplasia arising in the remnant esophagus after cardia resection. Gastrointest Endosc.

[62] Gatenby PA, Ramus JR, Caygill CP, Shepherd NA, Watson A (2008). Relevance of the detection of intestinal metaplasia in non-dysplastic columnar-lined oesophagus. Scand J Gastroenterol.

[63] Kelty CJ, Gough MD, Van Wyk Q, Stephenson TJ, Ackroyd R (2007). Barrett's oesophagus: intestinal metaplasia is not essential for cancer risk. Scand J Gastroenterol.

[64] Paris Workshop on Columnar Metaplasia in the Esophagus and the Esophagogastric Junction, Paris, France, December 11–12 2004. *Endoscopy* 2005, 37(9):879–920. 10.1055/s-2005-870305 16116544

[65] Long JD, Orlando RC (1999). Esophageal submucosal glands: structure and function. Am J Gastroenterol.

[66] Meining A, Bajbouj M (2010). Erupted cysts in the cervical esophagus result in gastric inlet patches. Gastrointest Endosc.

